# What kind of seed dormancy occurs in the legume genus *Cassia*?

**DOI:** 10.1038/s41598-020-69215-4

**Published:** 2020-07-22

**Authors:** Ailton G. Rodrigues-Junior, Marco T. A. Santos, Julia Hass, Bárbara S. M. Paschoal, Orlando C. De-Paula

**Affiliations:** Instituto de Biologia, Universidade Federal de Uberlândia, Uberlândia, 38405-320 Brazil

**Keywords:** Ecophysiology, Plant sciences, Plant ecology, Plant physiology

## Abstract

*Cassia* is a diverse legume genus widespread in the (sub-)tropical zone of the world. Several studies have been done on this genus; however, significant changes have occurred at the taxonomic level over the years. This has led to inaccurate information about seed dormancy in *Cassia* since many species are no longer included in the genus. Thus, our work aims to investigate and update the information about the kind of dormancy that occurs in seeds of *Cassia* species and also look into two notorious species in this group (*C. fistula* and *C. javanica*) to compare myxospermous vs. non-myxospermous seeds regarding dormancy and germination traits. Seed dormancy reports were found for 53 *Cassia* species, and the only kind of seed dormancy found for these species was physical dormancy (PY). Non-dormancy was not found, and all seeds had a blockage to water uptake during the dormant state, that is, all have PY. Of these 53 species, only 18 are currently included in the genus *Cassia*. *C. fistula* and *C. javanica* have fully developed embryos, and dormancy is only conferred by the (water-impermeable) seed coat. The lens in the seed coat is the only structure that creates a water pathway to break PY in *C. fistula*. Myxospermous seeds came out of dormancy faster than non-myxospermous ones. PY seems to be the only kind of seed dormancy that has evolved in *Cassia*. The extent of this kind of dormancy in all subtribe Cassiinae is also discussed.

## Introduction

*Cassia* L. sensu stricto was first proposed by Irwin and Barneby^1,2^, who segregated *Cassia *sensu lato into three genera (*Cassia* L., *Chamaecrista* Moech. and *Senna* Mill.), establishing the subtribe Cassiinae (Leguminosae: Caesalpinioideae). The genus *Cassia* was considered as the largest genus in Caesalpinioideae^3,4^, but currently it is comprised of only about 30 species^5^. Their fruits usually are indehiscent (also divided by transversal septa), unlike those of *Senna*, which are typically dehiscent and without septa separating the seeds^2^. The woody indehiscent pods of *Cassia* are quite tough, as well as the seeds. Additionally, *Cassia* fruits are not fleshy, but with remnants of pulp. The presence of these fruit traits suggests that large mammals could disperse *Cassia* seeds. However, a possible absence of seed dispersers for some species leads to speculation that these indehiscent fruits were likely dispersed by megafauna that are now extinct. Janzen and Martin^6^ reported that some fruit traits are better related to extinct animals (seed dispersal anachronisms), since some tough fruits may not be consumed (and seeds dispersed) by smaller extant animals. The absence of dispersers affects seed fate^6^, not only because of the lack of dispersal but also due to the ability of dispersers to break over the tough fruits and release the seeds.

Concerning seed dormancy, Baskin and Baskin^7^ proposed a classification system that includes five classes (see Baskin and Baskin^7^), wherein dormancy is associated with the embryo or other seed components (e.g. the seed coat). Among these five classes of dormancy, physical dormancy (PY) is the kind often found in legume seeds. The occurrence of PY is reported to increase in seasonally dry environments^8,9^, where *Cassia* species also occur. Additionally, PY is the only kind of seed dormancy reported in the sister of *Cassia,* the genus *Senna*^9–14^. Thus, this information could be indicative of a high incidence of physically dormant seeds in *Cassia*. PY is characterized by the presence of water-impermeable layer(s) in the seed coat (or fruit)^15^, thus, seeds cannot absorb water unless the ‘water gap’ is open or the water-impermeable layer(s) removed (i.e., by seed scarification).

Several studies have investigated the seed biology of *Cassia* species, especially the germination traits^16–20^. However, a significant number of species previously classified as *Cassia* are currently included in *Senna* and *Chamaecrista*. Thus, assembling the literature data and updating the information for *Cassia* will enable a wide-view on seed dormancy in this widespread genus. In 1960, *Cassia* was considered one of the largest genera (ca. 600 species) of dicotyledonous plants, highlighting the importance of this plant group^3^. However, information on seed dormancy occurring in *Cassia* is scattered and in most cases inaccurate, since a high number of previously *Cassia* species are not currently included in this genus. Also, the recent studies on PY have shown the complexity of this kind of dormancy^21–25^ and an investigation on water-impermeable seeds in this genus may allow new advances in the evolution of this restrict coat-imposed dormancy. Thus, we addressed the following questions: (1) What is known about seed dormancy in *Cassia*? (2) How many of the six classes of seed dormancy [including non-dormancy (see Baskin and Baskin^7^)] have been reported in *Cassia* species? (3) If seeds of *C. fistula* L. and *C. javanica* L. are dormant, are the embryo fully developed and/or the seed coat water impermeable at seed maturity and dispersal? (4) If they have PY, what is(are) the water gap(s) in these seeds? (5) Since the genus *Cassia* has myxospermous seeds, could water-impermeable seeds take advantage of myxospermy?

## Results

### Literature review on seed dormancy in *Cassia*

**Figure 1:**
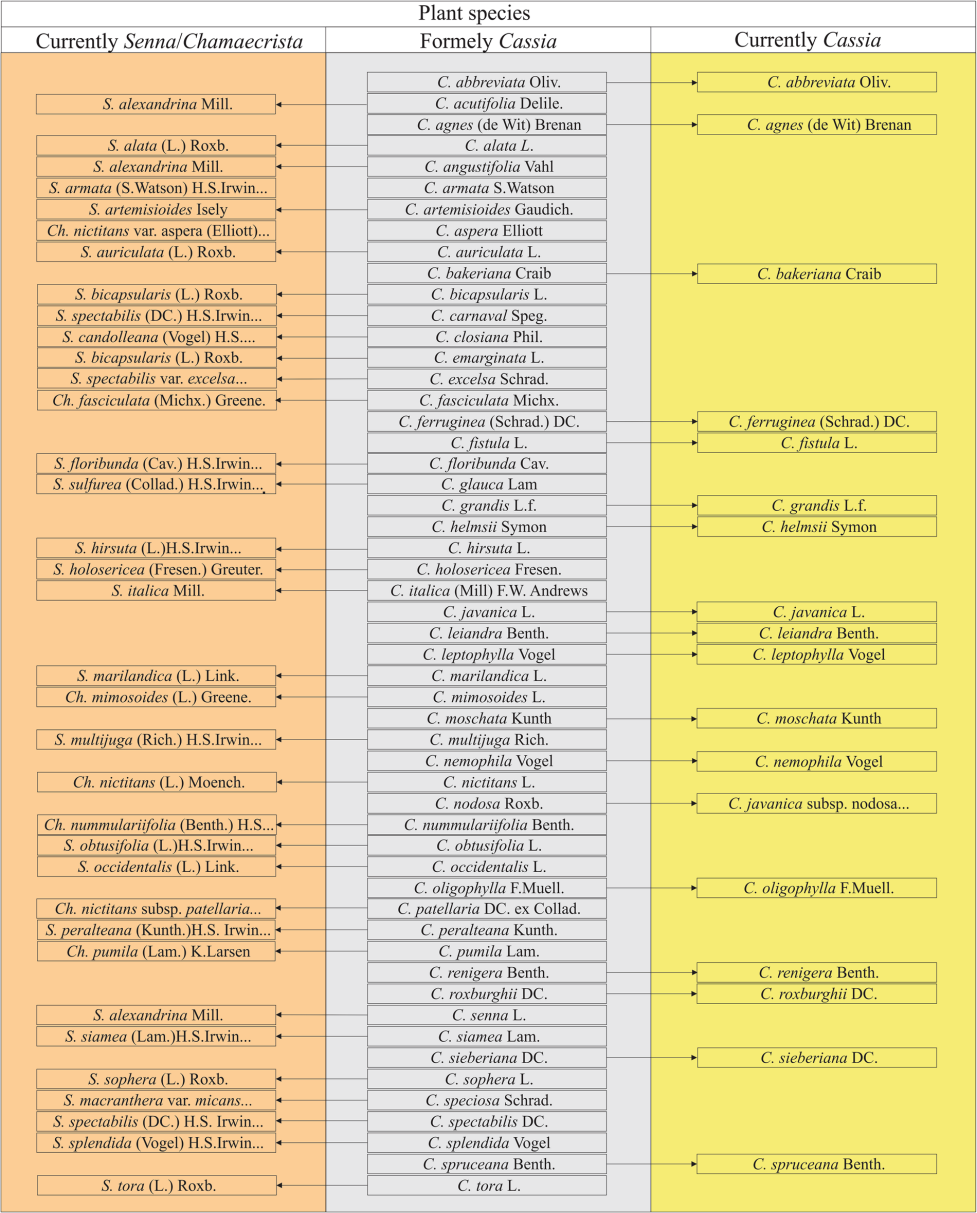
Overview of the changes in species included in the genus *Cassia*. All species included in this figure produce seeds with physical dormancy. Full species names: *C. javanica* subsp. *nodosa* (Roxb.) K. Larsen & S. S. Larsen; *Chamaecrista nictitans* subsp. *patellaria* (DC. ex Collad.) H. S. Irwin & Barneby; *Chamaecrista nictitans* var. *aspera* (Elliott) Torr. & A. Gray; *Chamaecrista* *nummulariifolia* (Benth.) H. S. Irwin & Barneby; *Senna* *armata* (S. Watson) H. S. Irwin & Barneby; *Senna* *candolleana* (Vogel) H. S. Irwin & Barneby; *Senna* *floribunda* (Cav.) H. S. Irwin & Barneby; *Senna* *hirsuta* (L.) H. S. Irwin & Barneby; *S. macranthera* var. *micans* (Nees) H. S. Irwin & Barneby; *Senna* *multijuga* (Rich.) H. S. Irwin & Barneby; *S. spectabilis* (DC.) H. S. Irwin & Barneby; *S. spectabilis* var. *excelsa* (Schrad.) H. S. Irwin & Barneby; *S. obtusifolia* (L.) H. S. Irwin & Barneby; *S. peralteana* (Kunth.) H. S. Irwin & Barneby; *S. siamea* (Lam.) H. S.Irwin & Barneby; *Senna* *spectabilis* (DC.) H. S. Irwin & Barneby; *S. splendida* (Vogel) H. S. Irwin & Barneby; *S. sulfurea* (Collad.) H. S. Irwin & Barneby.

Fifty-three *Cassia* species were found in the literature whose kind of seed dormancy was investigated. Thirty-five of these species are no longer included in *Cassia*. That is, these species have been transferred to *Senna* or *Chamaecrista* (Fig. 1). From all studies found, 18 species were currently classified as *Cassia* (see Fig. 1). All 53 species in Fig. 1 have seeds with PY.

The detailed results for the seed dormancy reported for species currently included in *Cassia* are shown in Table 1. The studies found in the literature showed that the investigated seeds had dormancy, and the treatments used to break PY (i.e., mechanical and acid scarification, immersion in hot water) were efficient in triggering high percentages of seed germination. As examples, Jurado and Westoby^18^ found that intact seeds of *C. helmsii* Symon and *C. oligophylla* F. Muell. germinated at low percentages (< 20%), reaching high germination after mechanical scarification (max. of 90%). Mechanical and acid scarification also were effective in breaking the dormancy of *C. leptophylla* Vogel^26^ and *C. renigera* Benth.^27^ seeds. For *C. nemophila* Vogel, nontreated seeds germinated to low percentages; however, immersion in hot water broke dormancy^17^.

**Table 1:** Seed dormancy in genus *Cassia*.

Species	Dormancy	References
*C. abbreviata*	PY	Tietema et al.^82^
*C. agnes*	PY	Watkins^83^
*C. bakeriana*	PY	Baskin and Baskin^9^
*C. ferruginea*	PY	Martins et al.^84^
*C. fistula*	PY	The present work
*C. grandis*	PY	Lopes et al.^28^
*C. helmsii*	PY	Jurado and Westoby^18^
*C. javanica*	PY	The present work
*C. leiandra*	PY	Moreira and Moreira^85^
*C. leptophylla*	PY	De Paula et al.^10^
*C. moschata*	PY	Souza and Silva^86^
*C. nemophila*	PY	Grice and Westoby^17^
*C. oligophylla*	PY	Jurado and Westoby^18^
*C. renigera*	PY	Reddy^27^
*C. roxburghii*	PY	Jayasuriya et al.^11^ and Jaganathan^87^
*C. sieberiana*	PY	Gill et al.^16^
*C. spruceana*	PY	Knowles and Parrotta^88^

*PY* physical dormancy.

In some studies, imbibition tests were conducted. Lopes et al.^28^ showed that seeds of *C. grandis* L.f. do not absorb water without mechanical scarification. Also, De Paula et al.^10^ found that intact *C. leptophylla* seeds do not absorb water, unlike those that were exposed to a wet heat treatment. Indeed, this thermal treatment acts on *C. leptophylla* seeds forming an opening in the micropyle, which was the water gap for this species^10^. The results for *Cassia* species indicated that PY is recurrent for this genus (Table 1).

Furthermore, the former *Cassia* species, including *C. acutifolia Delile*,* C. alata* L., *C. angustifolia* Vahl.,* C. armata* S. Watson, *C. artemisioides* Gaudich., *C. aspera* Elliott, *C. auriculata* L., *C. bicapsularis* L., *C. carnaval* Speg., *C. closiana* Phil., *C. emarginata* L., *C. excelsa* Schrad.,* C. fasciculata* Michx., *C. floribunda* Cav., *C. glauca* Lam., *C. hirsuta* L., *C. holosericea* Fresen., *C. italica* (Mill.) F.W. Andrews, *C. marilandica* L., *C. mimosoides* L., *C. multijuga* Rich., *C. nictitans* L., *C. nodosa* Roxb., *C. nummulariifolia* Benth., *C. obtusifolia* L., *C. occidentalis* L., *C. patellaria* DC. ex Collad., *C. peralteana* Kunth, *C. pumila* Lam., *C. senna* L., *C. siamea* Lam., *C. sophera* L., *C. speciosa* Schrad., *C. spectabilis* DC., *C. splendida* Vogel, *C. tora* L., now are included in *Senna* or *Chamaecrista* (see Fig. 1) and exclusively produce seeds with PY (see Faruqi et al.^29^, Martin et al.^30^, Rizzini^31^, Bhatia et al.^32^, Zegers and Lechuga^33^, Daiya et al.^34^, Teem et al.^35^, Gill et al.^16^, Felippe and Polo^36^, Kay et al.^37^, Khan et al.^38^, Cissé^39^; Al-Helal et al.^40^, Elberse and Breman^41^, Bhattacharya and Saha^42^, Rodrigues et al.^43^, Capelanes^44^, Todaria and Negi^19^, Francis and Rodriguez^45^, Lezama et al.^46^, Agboola^47^, Baskin et al.^48^, Jeller and Perez^49^, Fowler and Bianchetti^50^, Bargali and Singh^51^, Dutra et al.^52^, Ellis et al.^53^, Baskin and Baskin^9^, Mishra and Bohra^54^, Rodrigues-Junior et al.^13^).

### Response to dormancy-breaking treatments

**Figure 2:**
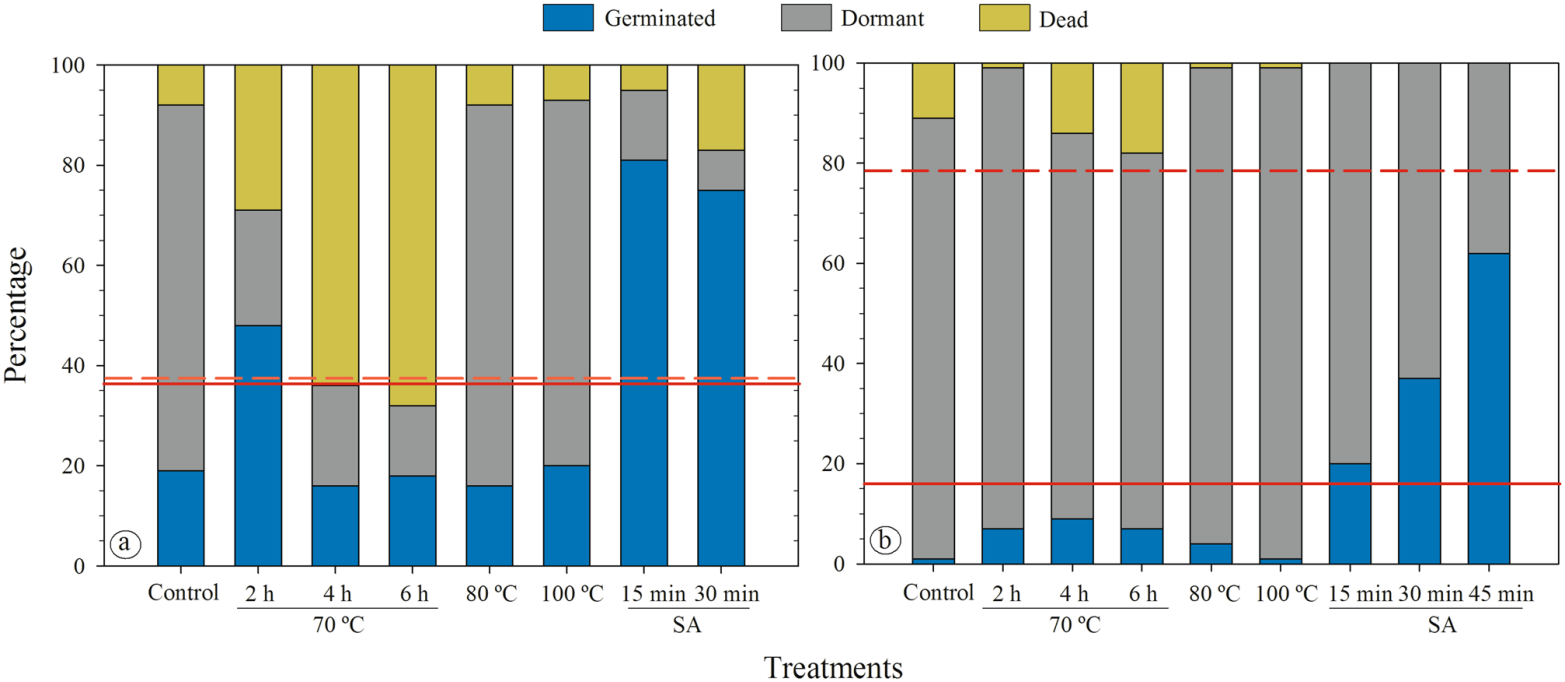
Dormancy, germination and mortality of *C. fistula* (**a**) and *C. javanica* (**b**) seeds subjected to different dormancy-breaking treatments. The solid red line indicates the mean of germination in all treatments. The dashed red line indicates the mean of dormant seeds in all treatments. *SA* sulphuric acid.

Seeds of *C. fistula* and *C. javanica* had distinct responses to the dormancy-breaking treatments (Fig. 2). The greater responsiveness of *C. fistula* seeds to the treatments compared to *C. javanica* was evident. Exposing *C. fistula* seeds to 70 °C (for 2 h) and sulphuric acid (15 min) increased the breaking of PY and consequently increased germination (Fig. 2a). However, an extension of the time seeds were exposed to each treatment increased mortality (Fig. 2a). For *C. javanica*, few seeds (1%) germinated without any treatment (19% for *C. fistula*), and thermal treatments were not efficient in breaking dormancy. Immersion of seeds in sulphuric acid for 45 min reached the highest germination (62%) for *C. javanica* (Fig. 2b). The mortality was not increased, and a high percentage of seeds was still dormant after being exposed to different treatments (Fig. 2b). Overall, *C. fistula* seeds germinated to higher percentages than those of *C. javanica* in all treatments (see solid red lines in Fig. 2). Additionally, the mean percentage of seeds that remained dormant following all treatments was low for *C. fistula* (37.6), while it was quite high for *C. javanica* (78.4) (see red dashed lines in Fig. 2). The high mortality for *C. fistula* seeds indicates that dormancy was broken, but the treatment damaged the embryo (Fig. 2a).

### Imbibition curve

**Figure 3:**
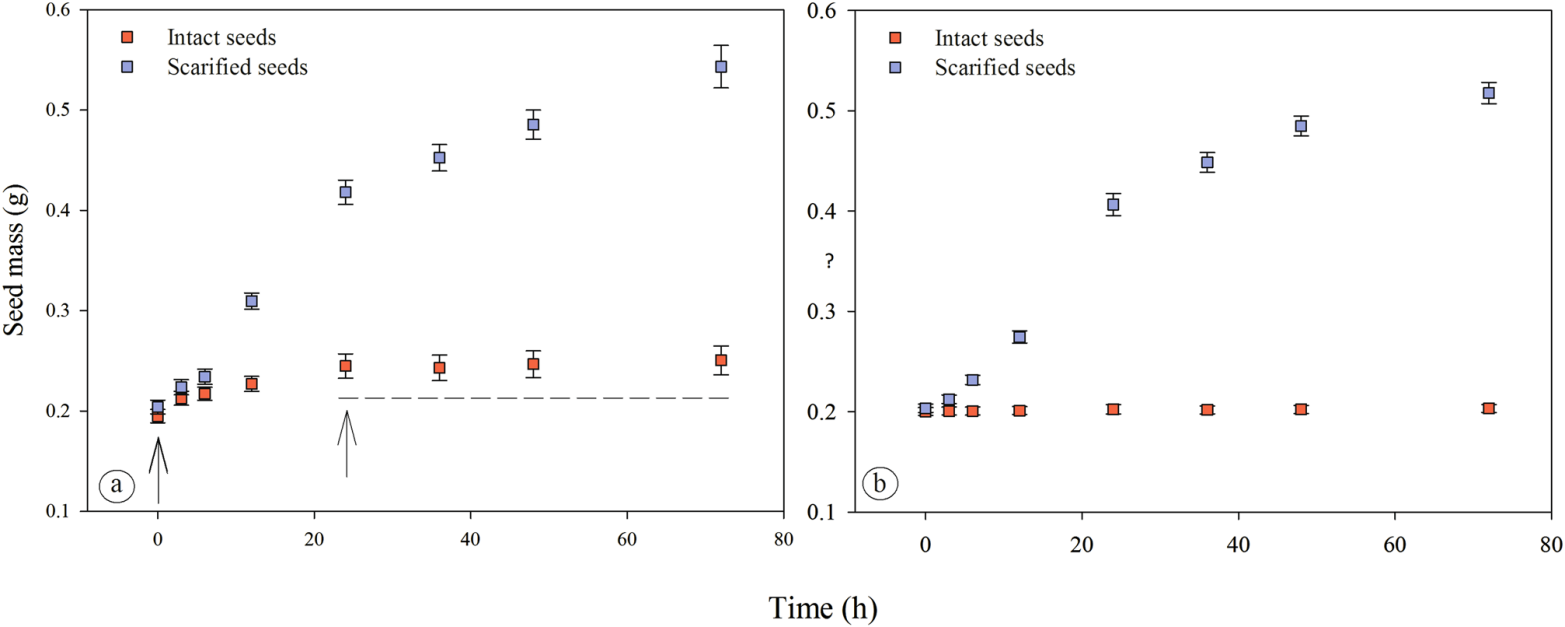
Changes in mass (mean ± SE) of intact and scarified seeds of *Cassia fistula* (**a**) and *C. javanica* (**b**). Arrows and dashed line in A indicate the increase in seed mass and subsequent stabilization, respectively. n = 30.

Seeds of both *Cassia* species increased in mass when placed in germination conditions following scarification (Fig. 3a,b). At 72 h of imbibition, seed mass of *C. fistula* and *C. javanica* had increased 166.4% and 154.5%, respectively. Concerning intact seeds, a distinct pattern was observed for *C. fistula*. There was a definite increase in seed mass (25.6%) until 24 h of incubation on a moist substrate for these seeds, and then stabilization afterward (Fig. 3a). This increase in mass of intact seeds was due to the thick mucilage on the seed coat. The mucilage absorbed water when the seeds were placed in germination conditions, and even after the seeds were blotted dry, the moisture remained adhered to the mucilage. After full hydration, the increase in seed mass ceased (Fig. 3a). On the other hand, there was no increase in seed mass of intact *C. javanica* seeds (Fig. 3b), showing a typical pattern of seeds with PY.

### Seed morphological traits

**Figure 4:**
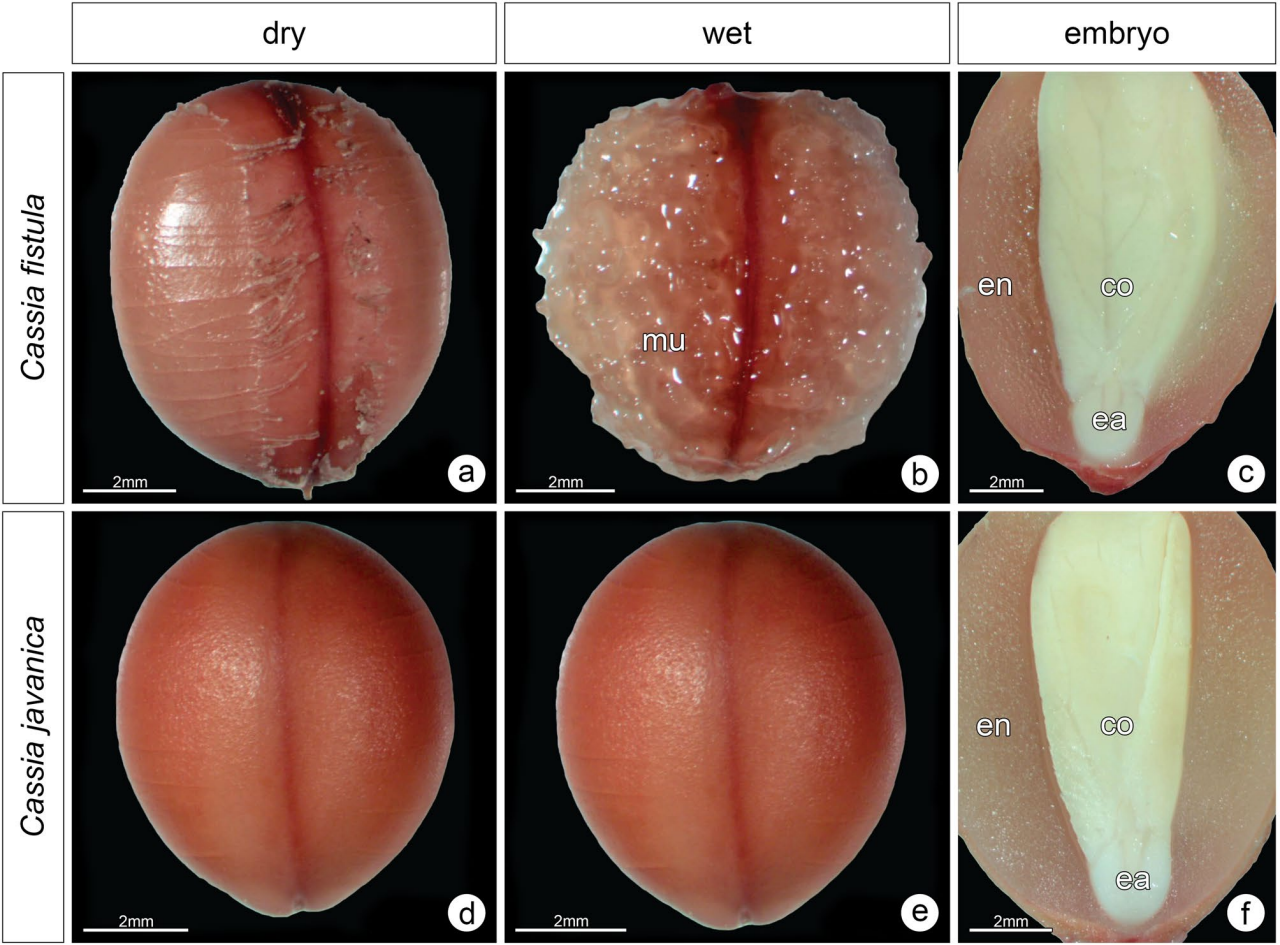
Morphological traits of *Cassia fistula* and *C. javanica* seeds. Dormant (water-impermeable) seeds under dry (**a**, **d**) and wet conditions (**b**, **e**), showing the release of mucilage in the myxospermous seeds of *C. fistula* (**b**). Longitudinal sections of seeds show the endosperm and the fully developed embryo with differentiated cotyledons and embryonic axis (**c**, **f**). *co* cotyledon, *ea* embryonic axis, *en* endosperm, *mu* mucilage.

Seeds of *C. fistula* had a rounded shape and a thick mucilaginous layer flaking off (Fig. 4a). Under moist conditions, *C. fistula* seeds released sticky mucilage that expanded and increased seed volume (Fig. 4b). In this assay, dormant seeds did not absorb water (except the mucilage), and only the mucilage expanded (Fig. 4b). Longitudinal sections of the seeds showed that the species had a thick endosperm surrounding the fully developed embryo (Fig. 4c). *C. javanica* seeds also had a rounded shape but with no evident mucilaginous layer (Fig. 4d). Under the moist condition, this seed did not produce mucilage; thus, it is characterized as a non-myxospermous seed (Fig. 4e). *C. javanica* also had an albuminous seed, with a thick endosperm surrounding the fully developed embryo (Fig. 4f).

**Figure 5:**
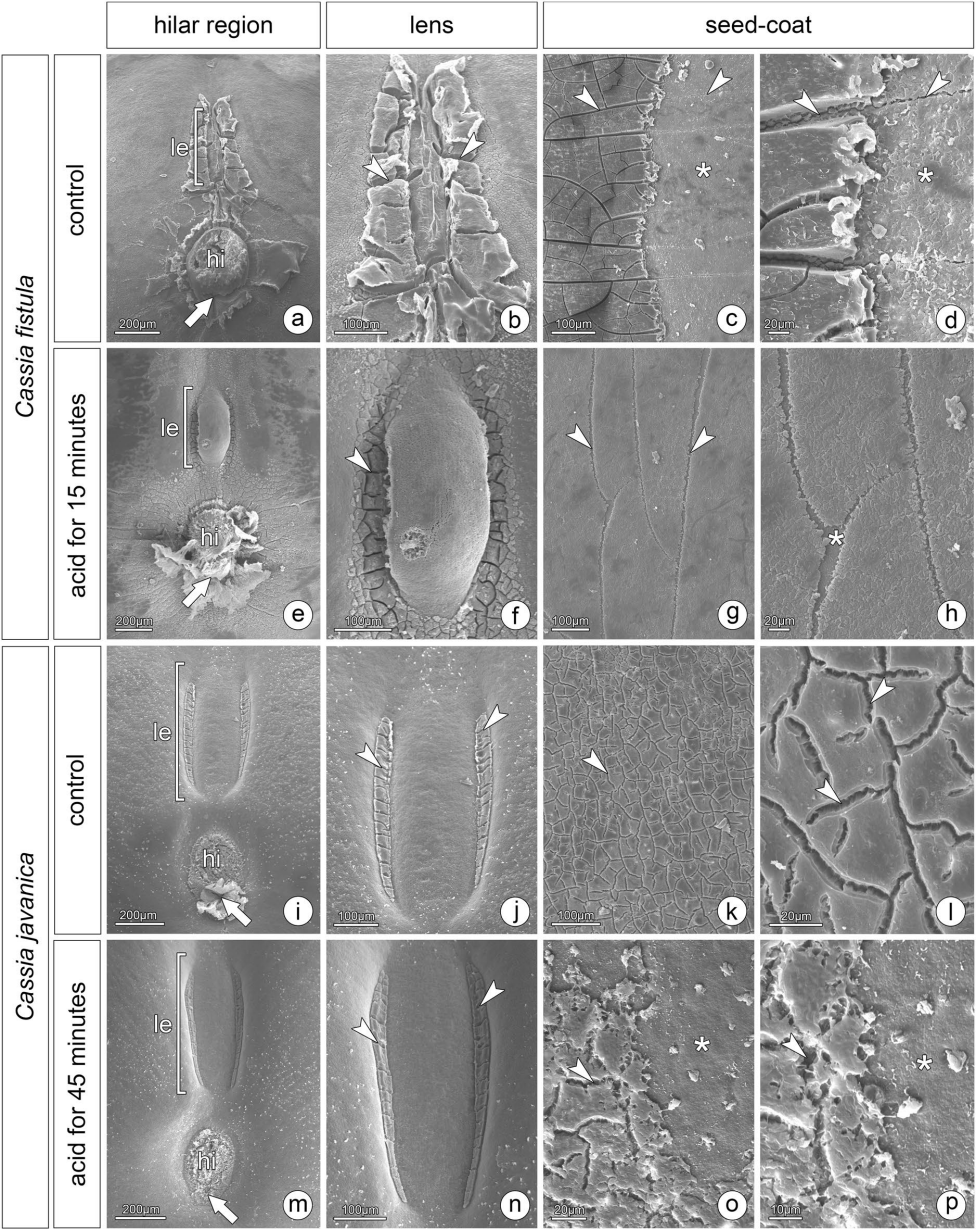
Morphological features of dormant and treated (nondormant) seeds of *Cassia fistula* and *C. javanica*. Hilar region of dormant *C. fistula* seed with a narrow lens, hilum and micropyle (arrow) (**a**, **b**). Extra-hilar region showing the seed coat flaking off (asterisk) and some cracks (arrowheads) (**c**, **d**). Treated seed with uplifted lens, and unchanged hilum and micropyle (arrow) (**e**). Lateral gaps formed in the lens (**f**). Fissures in the seed coat (**g**, **h**). Hilar region of dormant *C. javanica* seed with a lens, hilum and micropyle (arrow) (**i**). Detail of the lens with the lateral part sealed by a mucilaginous layer (arrowheads) (**j**). Cracks in the seed coat (**k**, **l**). Treated seed with unchanged structures (**m**, **n**) and partial removal of the mucilaginous layer (**o**, **p**). *hi* hilum, *le* lens.

*Cassia fistula* seeds had a hilar region composed of a narrow lens, hilum, and a tiny micropyle (Fig. 5a,b). The whole seed coat had several cracks but only in the mucilaginous layer (Fig. 5c,d). Following the dormancy breaking treatment, the lens was uplifted, creating a gap below it (Fig. 5e,f). The other structures did not change (Fig. 5e), and the seed coat lost the prominent cracks, due to partial removal of mucilage (Fig. 5g,h). Seeds of *C. javanica* had a hilar region with the lens, forming a wide depression on the coat, hilum and micropyle (Fig. 5i,j). The lateral part of the lens was sealed by a mucilaginous layer (Fig. 5j). The seed coat of this species also had cracks (Fig. 5k,l) but not prominent as in *C. fistula* (Fig. 5c,d). No evident structural changes occurred following dormancy breaking treatments (Fig. 5m,n), only partial removal of the mucilaginous layer (Fig. 5o,p). The absence of changes may be explained by the high percentage of dormant seeds even following treatments. The results were similar for both acid treatments (30 and 45 min).

## Discussion

### The kind of dormancy in the genus *Cassia*

The genus *Cassia* has changed drastically over time, from the largest genus in the Caesalpinioideae^3,4^ to a genus with about 30 species^5^. Among the 53 *Cassia* species for which we found dormancy studies in the literature, 66% of them are no longer included in this genus. However, all these studies report that PY was only kind of seed dormancy found PY. It is worth noting that the reports for the kind of seed dormancy cover 56% of the species in the genus *Cassia*. The control of seed germination in this genus is caused exclusively by the water-impermeable seed coat. All species found in the literature germinate promptly following dormancy-breaking treatments, and the embryos are fully developed at seed maturation. That is, seed dormancy is not related to the embryo. This coat-imposing dormancy is related only to the water impermeability of the testa, and the embryo is ready to start germination when the water enters through the water gaps located on the seed coat^15,55^. Our results showed the lens is the water gap in *C. fistula* seeds. This seed structure is the most common water gap in Leguminosae^56^. This structure was uplifted when PY was broken as shown for other species^13,14,57^, thereby creating an entry point for water into the seed. For *C. javanica*, the seed structure functioning as a water gap remains unclear.

Since seeds with PY do not imbibe water, the imbibition test is an accurate way to identify this kind of dormancy in seeds^9^. However, specific seed traits such as the presence of mucilage can hinder this detection. The increase in mass of myxospermous seeds is due to absorption of water by the mucilage^58, 59^. Thus, attention must be paid to imbibition tests performed using this type of seeds.

### Physical dormancy and myxospermy

To release PY, seeds must experience specific environmental conditions^21^. For some species, moist conditions in the environment are required for the breaking of PY^60,61^. Thus, in this case, physically dormant seeds overcome dormancy in moist soil^24,62^. Since seeds with PY can germinate promptly when the dormancy is released^9^, the strategy to break PY under moist conditions for some species allows seeds to germinate in a suitable environment for seedling establishment^24,62^. Thus, soil humidity is a key factor modulating the germination timing of some water-impermeable seeds. In this way, studies have investigated this water-dependent mechanism to break PY and related it to the increasing internal vapour pressure in the seed caused by the association between humidity and high temperatures, which dislodge the weak regions in the seed coat forming the water gap (see Rodrigues-Junior et al.^25^ and Jayasuriya et al.^63^).

Hence, if the moisture surrounding the seeds is essential for completing the dormancy breaking process for some species, the capacity of seeds to retain water next to the seed coat would facilitate this process. Thus, myxospermy could have an ecological significance for seeds with PY. Myxospermous seeds release mucilage when in contact with water, and the chemical composition of the mucilage makes it hydrate quickly, forming a hydrogel component that holds significant amounts of water surrounding the seed^64^. Mucilage can also be considered a ‘superabsorbent hydrogel’, due to its strong ability to absorb water^58^. The roles of myxospermy on seed dispersal and germination are detailed in the literature^59,64–67^. In contrast, the role of mucilage on dormancy is poorly investigated; however, if moisture is a crucial factor on releasing PY, myxospermy may affect this process in water-impermeable seeds. As demonstrated for the myxospermous and non-myxospermous seeds of *Cassia*, the species that produces mucilage broke PY easily in all treatments they were subjected to when compared to the non-myxospermous species.

Our results may stimulate in-depth investigations about a new possible role of myxospermy. Detailed investigations need to be done, such as removing mucilage before incubating the seeds under conditions that promote dormancy release. Mucilage-producing seeds are mostly found in phylogenetically advanced groups^59^, with some of them also producing water-impermeable seeds (i.e., with PY), for example *Guazuma ulmifolia* Lam.^22^, *Senna alata* (L.) Roxb.^13^ and the studied species *C. fistula*. Thus, this advanced evolutionary trait (mucilage), with ecological roles on several stages of the plant life cycle^59^, may also have a direct role on PY.

### Seed dormancy in the subtribe Cassiinae

The subtribe Cassiinae (comprising the genera *Cassia*, *Chamaecrista*, and *Senna*) has widespread members, and investigations about the kind of dormancy in their seeds have been done in several parts of the world. For *Senna*, the largest genus in this subtribe, all reports in literature showed that PY is the only kind of dormancy found in this group^9–12,14^. No results were found associating PY with another kind of dormancy, for example, with physiological dormancy (combinational dormancy). In the case of *Chamaecrista*, the second largest genus in this subtribe, the investigations also found PY as the only kind of dormancy occurring in these seeds^68–72^. The results found in the present study also corroborate with the evidence that impermeability to water is a prevailing trait in seeds of this subtribe, since PY was found in all *Cassia* species. All 53 species reported in our study, including those that are currently included in *Chamaecrista* or *Senna*, have PY.

All considerations about the evolutionary aspects of seed dormancy declare PY as the most recent germination constraint in the evolution of seed plants^7,73,74^. This advanced dormancy is restricted to certain groups of plants^73,74^ and occurs in 18 angiosperm families^22^. Even with the high diversification presented by the species included in Cassiinae^1,75,76^, all information here leads us to consider this restricted kind of seed dormancy as the only one that has evolved in this legume subtribe. This subtribe with PY is then adapted to specific environmental cues that make the seeds overcome water impermeability and be able to germination as soon as the water is available.

## Materials and methods

### Literature review on seed dormancy in *Cassia*

The purpose of this investigation was to identify the kinds of seed dormancy reported for the genus *Cassia*. Literature data were retrieved from published studies on seed dormancy and germination of *Cassia* species. Information found in the seed book by Baskin and Baskin^9^ was also used, and the original papers searched to check the full plant names. Since several species previously included in *Cassia* were transferred to *Senna* or *Chamaecrista*, or it was updated, all species names were checked individually in *The Plant List* (https://www.theplantlist.org/) and *Flora do Brasil* (https://floradobrasil.jbrj.gov.br/) for an update on seed dormancy in this legume genus.

### Seed dormancy in two *Cassia* species

*Cassia fistula* L. and *C. javanica* L. are two introduced species to Brazil^77^ and two of the most widely cultivated plants of the genus^5^. Fruits of *Cassia fistula* (N-18.883391 S-48.260003) and *Cassia javanica* (N-18.889629 S-48.282410) were collected at Uberlândia (Southeastern Brazil) before dispersal, when the fruits were dry on the mother plant. Mature fruits of both species remain attached to the mother plant for several months, and the seeds are not released from them. These two species are trees and grow in Brazilian savanna (Cerrado), characterized by two well-defined seasons throughout the year: the dry season, during the autumn/winter; and rainy season, during the spring/summer^78^. Since the fruits are lignified and challenging to open manually, the seeds were removed using a hammer to break the fruits. To remove non-filled seeds, they were separated by flotation in the water, especially for *C. fistula*, whose seeds have a high predation percentage. Seeds were blotted dry and placed in plastic trays at ambient room conditions (25 ± 5 °C) for 24 h and then stored in paper bags until the beginning of the dormancy and germination experiments, one week later.

### Response to dormancy-breaking treatments

*Cassia fistula* produces myxospermous seeds, while *C. javanica* produces non-myxospermous seeds. Thus, the purpose of this experiment was to compare the response of both species to different treatments to break dormancy. The seeds were subjected to the following treatments: (I) immersion in hot water at 80 °C for 15 min^12,13^; (II) immersion in boiling water (100 °C) for 5 s.; immersion in sulphuric acid (96%) (III) for 15, (IV) 30, and (VI) 45 min; thermal treatments at 70 °C in moist conditions (seeds placed on moistened germination paper in a sealed Gerbox and incubated in an oven) for (VII) 2, (VIII) 4, and (IX) 6 h. Preliminary tests showed that dry treatments were not effective in breaking dormancy for the tested seeds. Seeds not subjected to any treatment were considered the control group. To evaluate germination, seeds were placed in gerboxes on germination paper moistened with distilled water and incubated at 25 °C under constant light (cool white fluorescent tubes, 40 µmol m^−2^ s^−1^). Four replications of 25 seeds were used for each treatment. Germination was monitored at 3-day intervals for 30 days, and the criterion for germination was the protrusion of the radicle.

### Imbibition curve

Changes in mass were quantified for intact and scarified seeds of *C. fistula* and *C. javanica* to verify the presence (or not) of a water-impermeable seed coat. Thirty intact and manually-scarified seeds (using sandpaper) for each species were placed in gerboxes on germination paper moistened with distilled water. Seeds were placed in a sequential order in each Gerbox (30 seeds per Gerbox) and weighed individually. If the seeds are water-impermeable, scarification will remove the restriction to water uptake, resulting in increased seed mass when in contact with water. Seeds were incubated at 25 °C under constant light and weighed consecutively using a precision scale. Seeds were blotted dry before each weighing and weighed at distinct intervals for 72 h.

### Seed morphological traits

Presence (or not) of mucilage, seed coat structures (water gap), and embryo development of *C. fistula* and *C. javanica* seeds were evaluated under a stereomicroscope and scanning electron microscope (SEM). First, seeds were evaluated under a stereomicroscope following exposure to dry and moist conditions. Under moist condition, seeds were placed on moistened paper in a Gerbox for 24 h, while under dry conditions the paper was not moistened. Mucilage in myxospermous seeds expands upon hydration, and the differences between the species were examined. Seeds were also manually scarified and imbibed for a few minutes and then longitudinally sectioned using a cutting blade to check if the embryo was fully developed at seed maturity. Additionally, intact and treated (according to the breaking dormancy methods used above) seeds of *C. fistula* and *C. javanica* were fixed in Trump fixative for 24 h^79^, dehydrated in an ethanol series and subjected to critical point drying. Seeds were then mounted on stubs using double-sided carbon tape and sputter-coated with gold^80^. The whole seed surface was examined under SEM to investigate the structural changes on the seed coat.

### Statistical analyses

The experimental design was completely randomized. Germination data were analysed using a generalized linear model (GLM) followed by LSD test to compare the means at 5% probability^81^. The graphs were designed using the software Sigmaplot (Systat Software, San José, CA, USA).

## Data Availability

All data generated in this study are included in the main text.
